# The Vaults of Umm al-Dabadib: Geometric Study

**DOI:** 10.1007/s00004-020-00532-x

**Published:** 2020-10-22

**Authors:** Corinna Rossi, Fausta Fiorillo

**Affiliations:** Dipartimento di Architettura, Ingegneria Delle Costruzioni e Ambiente Costruito, Politecnico di Milano, Via Ponzio 31, 20133 Milan, Italia

**Keywords:** Nubian vaults, Elliptical vaults, Geometric constructions, Late roman forts, Kharga oasis, Ancient egypt

## Abstract

This article focuses on the shape of the vaults that cover the rooms of the Fort of Umm al-Dabadib (Kharga Oasis, Egypt’s Western Desert), dating to the Late Roman Period. This building is the central element of the contemporary Fortified Settlement, consisting of a dense, three-dimensional mosaic of domestic units, all covered by similar vaults, and belonging to a chain of similar installations. Two elements make Umm al-Dabadib an interesting case-study: the excellent preservation of its architectural remains, and the possibility to rely on an accurate photogrammetric survey of the entire built-up area. Thanks to this combination, it was possible to analyse the geometric shape of the vaults in connection to the ancient building techniques. The study determined that the vaults of the Fort are elliptical; this conclusion will impact on the study of all the similar settlements built in the Kharga Oasis, and possibly to other contemporary buildings elsewhere in Egypt.

## Introduction

Reliable geometric and metrological studies can only be performed on data derived from precise and accurate architectural surveys. Range- and image-based survey techniques, such as laser scanner and photogrammetry, make it possible to record the shape of an object (a building, in this case) to an automatically greater accuracy in comparison to the results of hand-made surveys. In particular, the three-dimensional models that can be generated from the survey data capture the actual shape and dimensions of the various architectural elements without the potential distortions due to the subjective perception of the surveyor and the personal interpretation of the draughtsman.

**Fig. 1:**
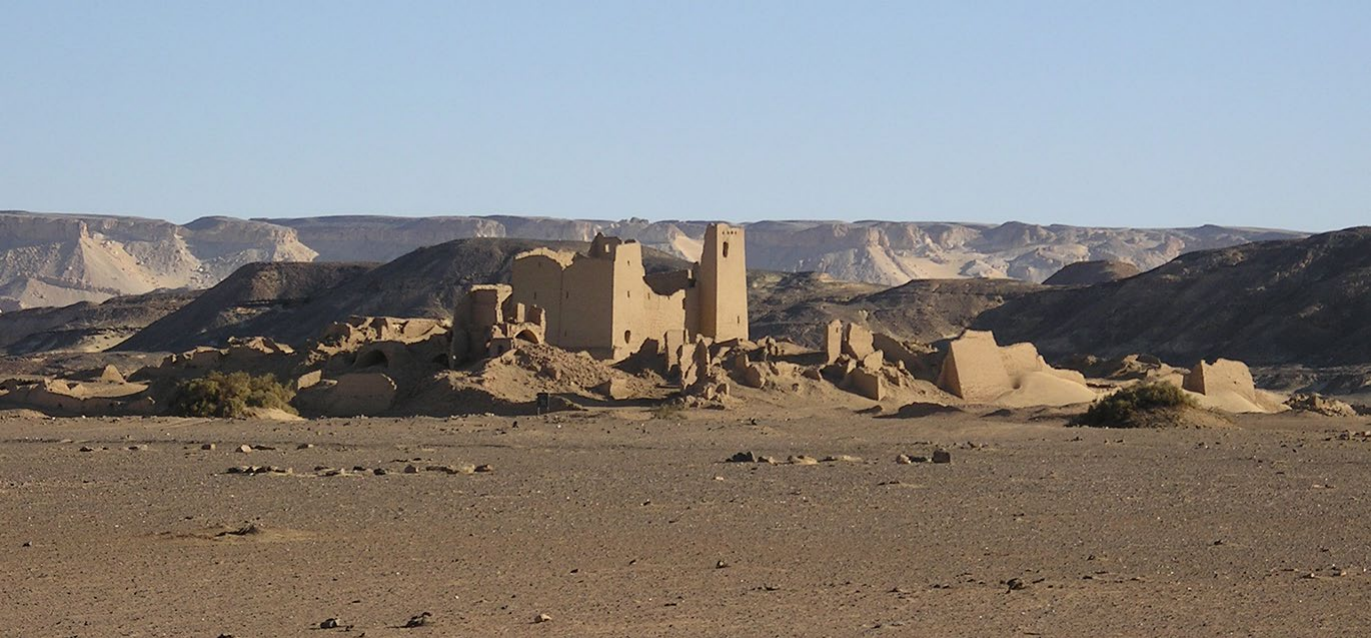
View of the Late-Roman settlement of Umm al-Dabadib, Kharga Oasis, Egypt’s Western Desert (Photograph 2013 by C. Rossi)

Geometric and metrological analyses are not merely formal exercises: they can offer precious information on the planning and building techniques and on the cultural and technical background of the builders. The Late Roman archaeological site of Umm al-Dabadib, located at the outskirts of the Kharga Oasis, in Egypt’s Western Desert (Fig. 1), represents a significant chance to carry out such analyses to improve our knowledge of this site, its background and its history.

The site, noted at the beginning of the twentieth century (Ball 1900; Beadnell 1909), was investigated in 1998 (Rossi 2000) and studied in detail for the first time in 2001–2003 (Rossi and Ikram 2018: Ch. 6). The photogrammetric survey carried out in 2014–2015 (Fassi et al. 2015) provided a reliable basis for further studies carried out within the ERC-funded LIFE programme, aiming at identifying a set of criteria for studying Late Roman settlements located along the desert frontier. The analysis of the data (Fiorillo and Rossi 2017) and the ensuing metrological analysis of the central Fort demonstrated that this building, constructed in the early fourth century AD as part of the Roman strategy of control of the Western Desert (Rossi 2013, 2018: 433–434, 449–451), was planned and built using the ancient Egyptian unit of measurement, the cubit (Rossi and Fiorillo 2018).

The implications of this discovery are manifold. First of all, it moved three centuries forward the latest attestation of the use of the ancient Egyptian cubit in architecture. Next, it revealed that the building and planning process were in the hands of a local workforce. Finally, a careful extension of the discussion to the other contemporary forts that punctuate the Kharga Oasis revealed that they, too, were built using the same unit of measurement, thus suggesting that the impact of these results should be extended to a regional scale (Rossi 2019).

**Fig. 2:**
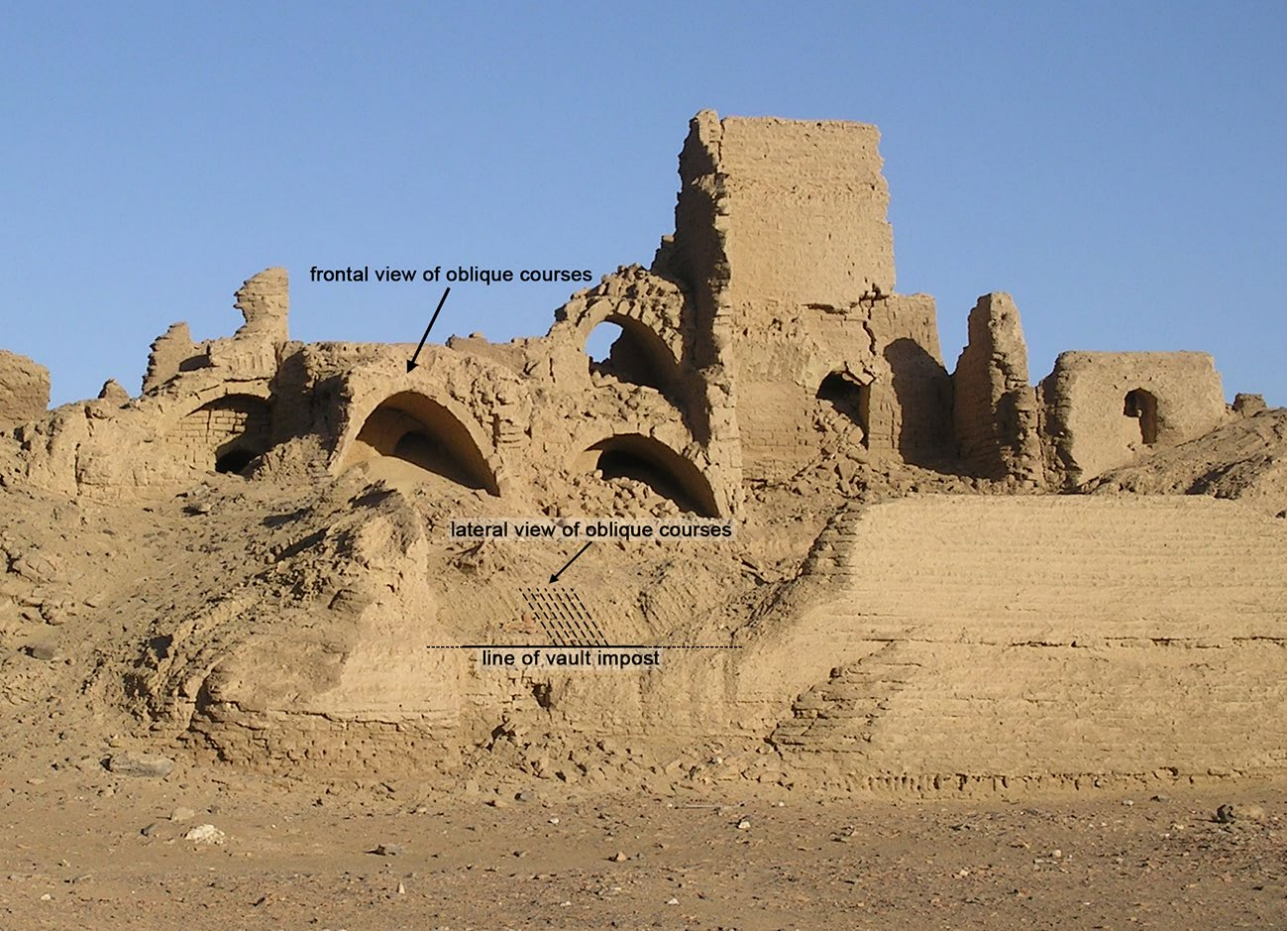
Portion of the fortified settlement of Umm al-Dabadib seen from east (Photograph 2013 by C. Rossi)

A parallel line of investigation is presented here: the Late Roman buildings to be found in Kharga (as well as in other oases) are characterized by the ubiquitous presence of barrel vaults that cover the vast majority of the rooms of houses, forts and magazines (Fig. 2). This article will focus on the geometric analysis of these vaults: it will prove that their profile was elliptical and that this shape corresponded to a simple and efficient construction technique.

## Names and Shapes

**Fig. 3:**
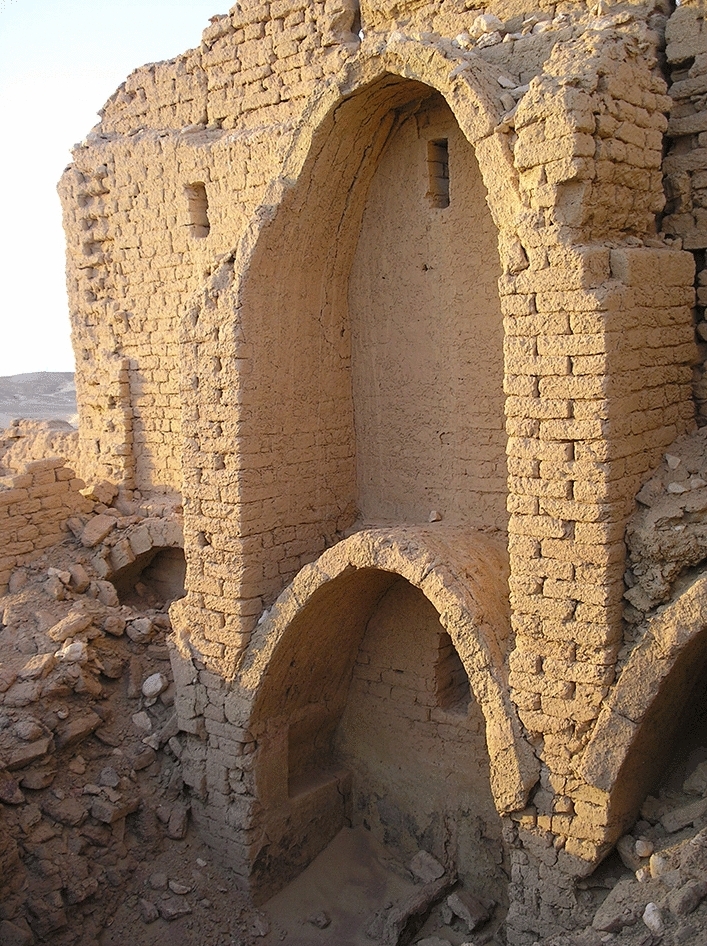
Remains of the vaulted rooms along the northern wall of the Fort of Umm al-Dabadib (Photograph 2013 by C. Rossi)

The most common type of vault to be found in the region is generally called ‘Nubian’, as it is typical of twentieth-century Nubian architecture (Zabrana 2018); however, this type of vault has been used in Egypt since the Old Kingdom (Kemp 2000: 93–96; see also Arnold 2003) and over the centuries has spread to all arid and semi-arid regions, where timber is scarce (Granier et al. 2006; also Van Beek 1987). The basic characteristic of this vault is that it can be constructed without any supporting system (centring or formwork) (Wendland 2007), as it consists of a succession of oblique arches leaning on the back wall of the room to be roofed over, made of bricks laid edge-to-edge along their short side (Fig. 3). The technical name of this constructional solution is ‘pitched-brick (barrel) vault’ (Lancaster 2009, 2012: 18).

The profile of the vault, that is its vertical section, generally corresponds to a more or less ‘elongated’ arc. In the specific case of ancient Egyptian vaults, Alan Spencer (1979: 124) hinted that the profile of the vaults corresponded to a catenary; the few examples that have been studied from a geometric point of view, however, appear to be elliptical or to correspond to segments of circles (Rossi 2004: 114–117). Concerning ancient Roman architecture, the shape of the arches has been generally described as “parabolic, semi oval or catenary” (Lancaster 2015: 49).

According to Lynne Lancaster, “in reality, the precise geometry for these elongated vaults is inconsequential because the form in most cases was simply derived from the initial construction process, rather than having any theoretical basis” (2015: 49). In the modern construction technique adopted in Sahel, the change in profile is achieved empirically (Granier et al. 2006: 9) by designing a curve that cannot be traced back to a precise geometric shape; in these cases, the buildings consist of one level only, which is not the case of the Fort of Umm al-Dabadib, as we shall see below.

**Fig. 4:**
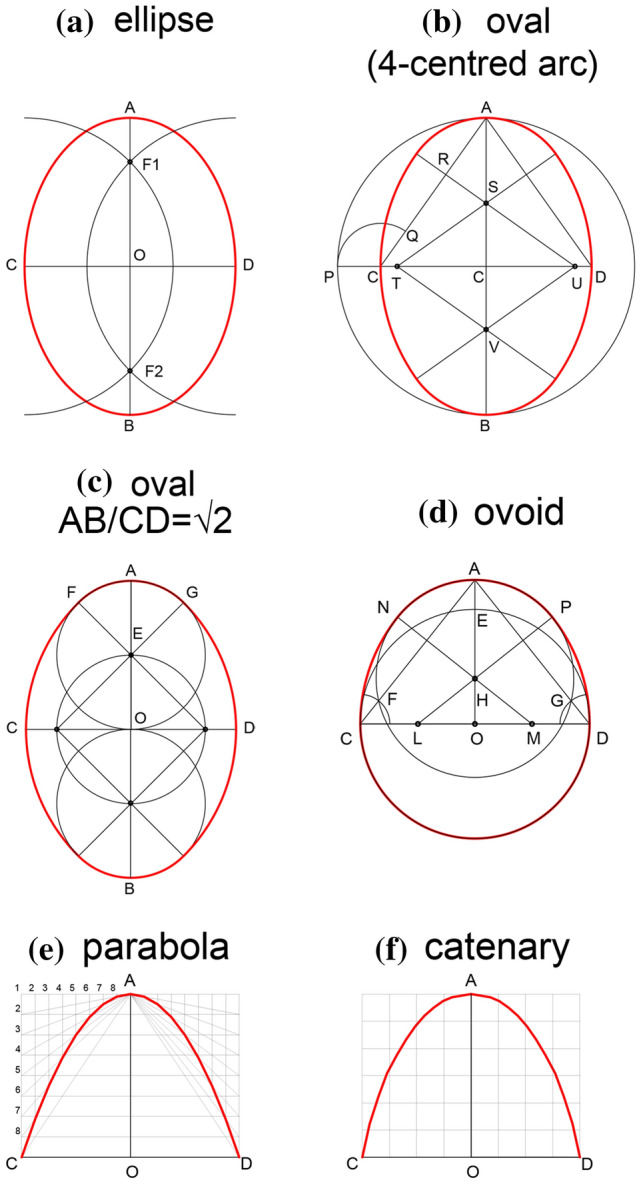
The array of the curves taken into account: **a** ellipse; **b**, **c** oval; **d** ovoid; **e** parabola; **f** catenary (drawing by F. Fiorillo)

The research question lying at the basis of this article is whether or not the resulting curve corresponded to a precise geometric shape, and if so, which shape had been used. We took into account four curves; for one of these, however, we considered three geometric constructions, thus bringing the total number to six (shown in Fig. 4).

In the graphic convention chosen for Fig. 4, the major axis is set vertically, in the same direction in which the vaults were built; the vaults, of course, correspond to the ‘upper halves’ of these curves.

The first is an ellipse, a curve in which the sum of the distances between each point and the two *foci* is constant (Fig. 4a). The second corresponds to a four-centred curve (an oval), made of portions of circumferences (sharing a tangent at their connecting points) symmetrically placed along the two axes (Fig. 4b); when the proportion between the two axes of an oval is equal to √2, the curve can be drawn using a geometrically simple construction (Fig. 4c). Similar to the oval is the ovoid, another four-centred curve in which, however, one pair of circumferences (in this case those along the vertical axis) is not symmetrical (Fig. 4d). The adjective ‘parabolic’ is often used to vaguely indicate a tall and narrow arch, but from a strictly geometric point of view it refers to an open conic section (Fig. 4e). The definition of parabola is the geometric locus of the points of the plane equidistant from a point called the focus and from a straight line called the directrix. Therefore, the parabola runs on either side of an axis of symmetry, and the vertex is its only point intersecting the axis. If, in addition to the vertex and axis, a point on the plane is assigned, there is only one parabolic curve that has that vertex, that axis and that passes through that point. Finally, a catenary is the downward curve designed by a chain hanging down from its two extremes under its own weight (Fig. 4f); this curve, if transposed into an upward arch, is able to efficiently sustain itself. The catenary curve has a uniform distribution of its total weight at each point, it represents the configuration for which the potential energy is minimal at each point.

## The Corpus of Vaults

**Fig. 5:**
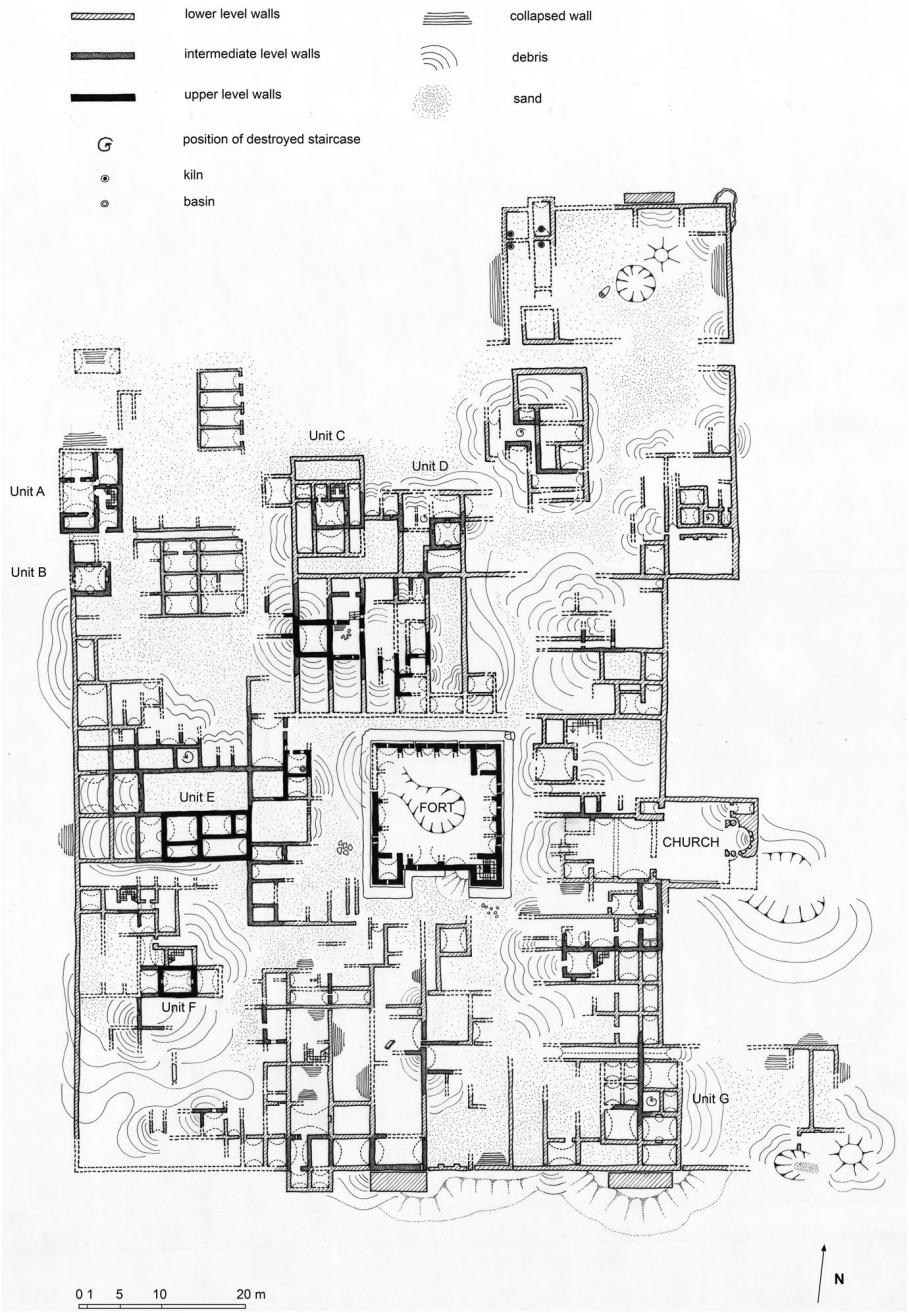
Plan of the fortified settlement of Umm al-Dabadib (drawing by C. Rossi)

The Fortified Settlement of Umm al-Dabadib consists of a dense built-up area measuring ca 80 × 110 m (Fig. 5). Most of the buildings appear to be domestic units distributed on two or three levels; all rooms were covered by pitched-brick barrel vaults. No open-air roads are visible, and it seems that the various blocks were served by long and narrow corridors, vaulted over using the same system.

From a constructional point of view, the barrel vaults covered rectangular rooms whose shapes range from nearly square to long and narrow. They always spring from the longer sides of the room and span over the shorter dimension. The lateral walls were generally 1 cubit thick (ca 52 cm) up to a certain height (Rossi and Fiorillo 2018: 382–383), then two opposite steps were created on either side of the room, from which the construction of the vault started. Thinner versions of these walls continued upwards until the upper floor level, where they re-gained their original thickness and continued along the same pattern. The lateral spaces between the vault and the vertical walls were filled by debris; in the Fort the builders used also roundish white stones as additional filling; many of these are accumulated in the now empty hollows on either side of the remains of the ruined vaults, as the thinner and more fragile filling was eroded and blown away by the wind over the centuries (see Fig. 3).

The oblique arches that progressively formed the vault were laid on a layer of mortar and each leaned on the previous one starting from the back wall, which was evidently built in advance. The mudbricks employed in the construction of the vaults were kept in position by either potsherds or, more often, by black stone chippings acting as wedges; although in some instances the mudbricks employed in the construction of vaults were different from those used for the walls, at Umm al-Dabadib normal mudbricks were widely also used to build vaults (see Warner 2018: 459–460).

**Fig. 6:**
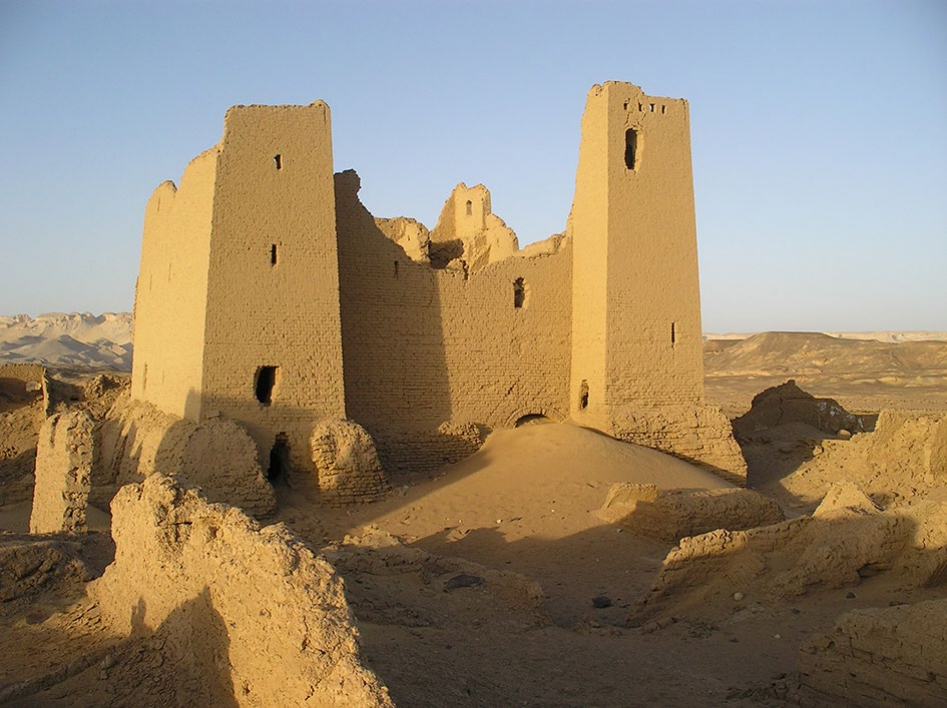
View of the Fort of Umm al-Dabadib from south (Photograph 2013 by C. Rossi)

**Fig. 7:**
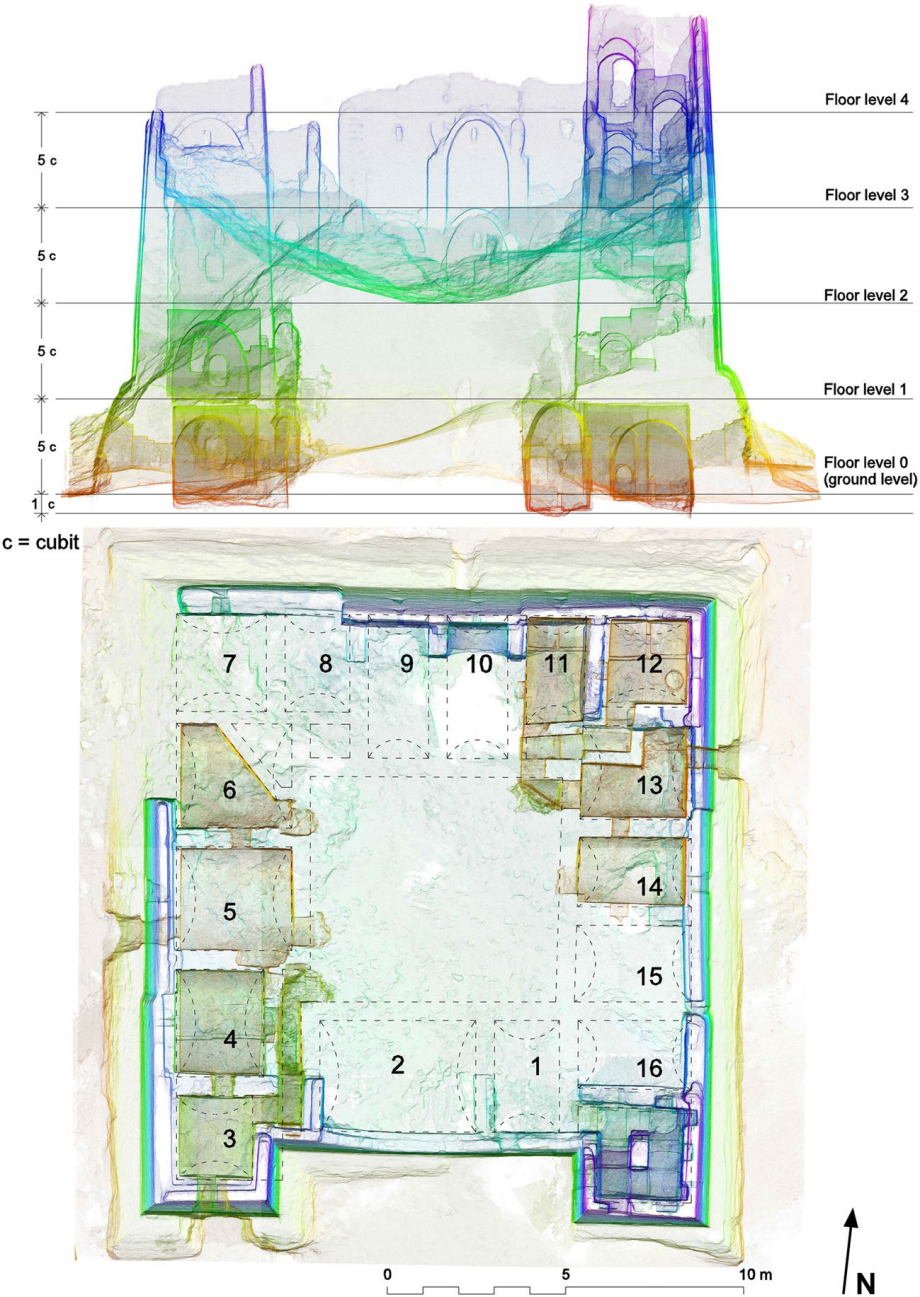
Top and frontal view of the three-dimensional point model of the Fort and numbering system adopted to identify the rooms (height of the color range corresponding to 60 cm, drawing by F. Fiorillo)

The central Fort (Fig. 6) consisted of five levels of rooms disposed along the four sides of an internal courtyard; all chambers were covered by pitched-brick barrel vaults. In fact, this building represents an excellent case study as a total of thirteen such vaults could be noted, surveyed and analysed. They have been labelled according to the level to which they belong (from 0 for the ground floor to 4 for the fifth level) followed by a number referring to their sequential position counted clockwise starting from the entrance (Fig. 7). In particular, the three-dimensional model of the Fort derived from the photogrammetric survey allowed to precisely measure the interior of ten rooms (eight at level 0 and two at level 1) and the partially collapsed remains of three further vaults on the south and the east side. Moreover, it is possible to reconstruct the profile of seven partially visible vaults, by interpolating the visible part of the existing profiles and referencing it to the metric information of their complete counterparts.

**Fig. 8:**
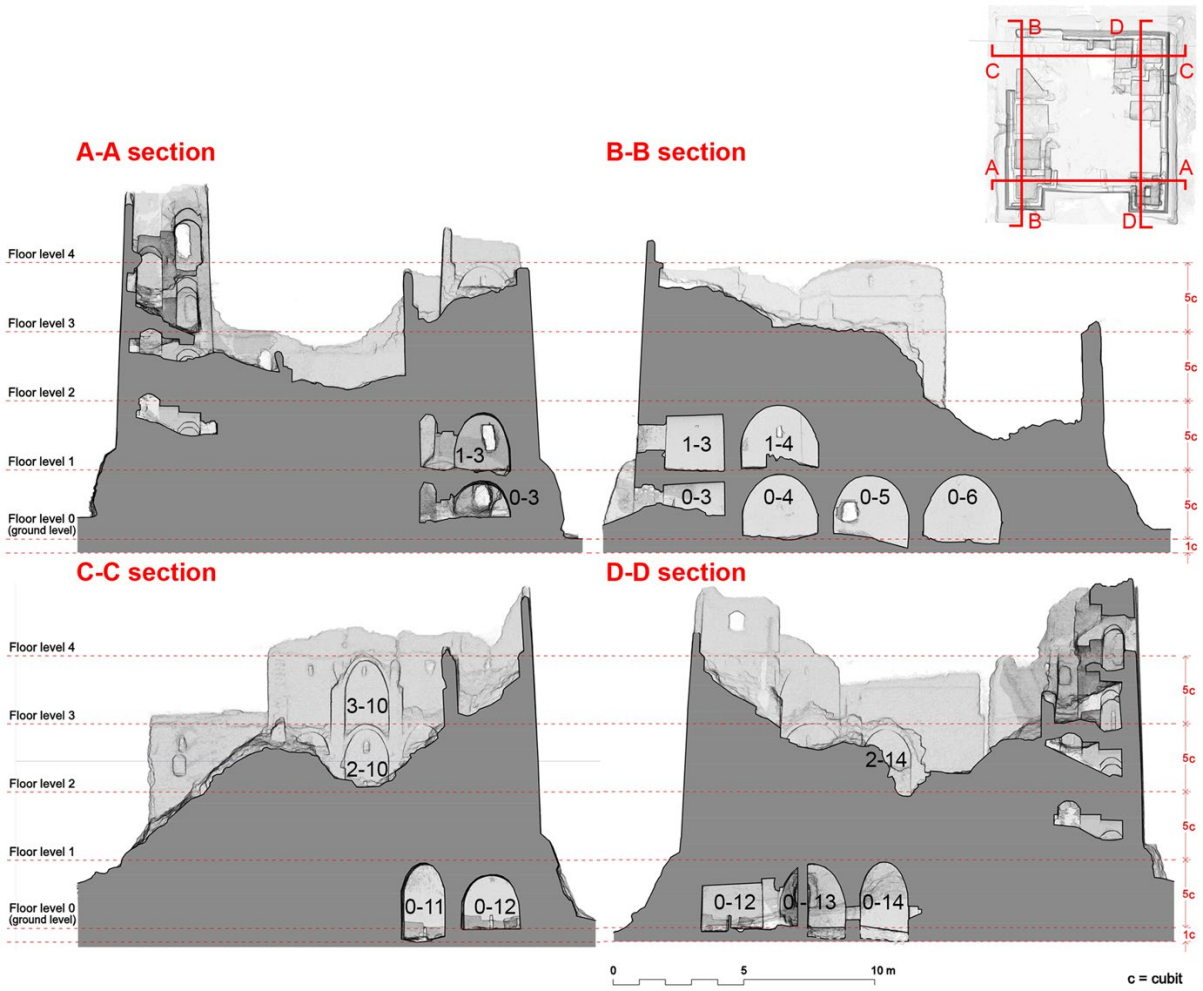
Vertical sections of the Fort of Umm al-Dabadib, cut near the external walls: A-A, looking southwards; B-B, looking westwards; C-C, looking northwards; and D-D, looking eastwards (drawing by F. Fiorillo)

For the purpose of this article, the Fort was cut along four sections, each running at a short distance from the external wall and looking towards it (Fig. 8). On the first section A–A, looking south, there are two vaults with a perfectly recognizable shape (0–3 and 1–3) and one with a partially visible profile (3–3). This is the most difficult section to be analysed because no metric information can be derived from and on the central part; moreover the two square towers, which contain the staircase to the east and a vertical sequence of rooms to the west, interrupt the rhythmic subdivision into similar rooms that characterises the other three sides of the building. On the B–B section, looking west, there are four visible vaults (0–4, 0–5, 0–6 and 1–4) and one partially visible, of which it is possible to assume the shape (2–5). On the C–C section looking north, there are four vaults with a perfectly recognizable shape (0–11, 0–12, 2–10 and 3–10) and three with a partially evident profile (2–7, 2–9 and 2–11). Finally, on the D–D section, looking east, there are three visible (0–13, 0–14 and 2–14) and 2 partially visible vaults (2–13 and 3–13). Table 1 contains the complete list.

**Table 1:** Visible and partially visible vaults of the Fort of Umm al-Dabadib

Section	Visible vaults	Partially visible vaults
A-A	2 (0–3, 1–3)	1 (3–3)
B-B	4 (0–4, 0–5, 0–6, 1–4)	1 (2–5).
C-C	4 (0–11, 0–12, 2–10, 3–10)	3 (2–7, 2–9, 2–11)
D-D	3 (0–13, 0–14, 3–14),	2 (2–13, 3–13)

## Retrieving the Shape

The target of our investigation was to identify a solution that would fit two criteria: be reliable from the point of view of the geometric correspondence to the actual vaults and feasible from a constructional point of view, that is, achievable thanks to a simple method to be easily adopted by the workmen.

From the previous metrological analysis of the Fort, we know that the distance between floor levels had been established as 5 cubits (2.6 m) (Rossi and Fiorillo 2018: 386; see also Fig. 7). This means that during the construction of each room and its corresponding vault the workmen started from two known data: the width of the room and the maximum height that they could reach (the apex of the extrados of the arch represented the reference point for the planking level of the upper floor, see Fig. 3). From a geometric point of view, this means that, for each vault, both the clear span and the rise were known; in this building the proportion between these two data, however, varied considerably. Therefore, the workmen could, in theory, choose among four geometric constructions: the elliptical arc, given the two diameters; the three-centred arc, given the two axes (Docci et al. 2017: 339–342); the parabola, given the vertex, the axis and a point (Docci et al. 2017: 58); and the catenary. In practice, however, the parabola can be immediately ruled out as it does not fit the two criteria lying at the basis of our investigation. First of all, in its middle sector this curve is significantly and consistently ‘narrower’ than the profile of the visible vaults. Secondly, a parabola cannot be easily constructed by means of ropes and nails: mathematicians such as Pappus of Alexandria (fourth century AD), a contemporary of the builders of the chain of Roman forts which includes Umm al-Dabadib, as well as the later Eutocius (sixth century AD) lamented the difficulty of drawing conic sections (Cuomo 2000: 191). Catenary curves, which were codified and distinguished from parabolas only in the seventeenth century, share the same problems (De Carlo 2019).

Instead, ovals and ellipses can be easily designed by means of ropes and nails. Their profile is very similar (Migliari 1995), to the point that when the length of their axes is the same they are difficult to distinguish (Duvernoy 2015: Fig. 2). For this reason, we tested four figures on the profile of the vaults: the ellipse and three three-centred arcs of differing proportions. Among the various geometrical constructions of polycentric ovals (Mazzotti 2019), we chose the ones in which the clear span and the rise (or their ratio) were known.

**Fig. 9:**
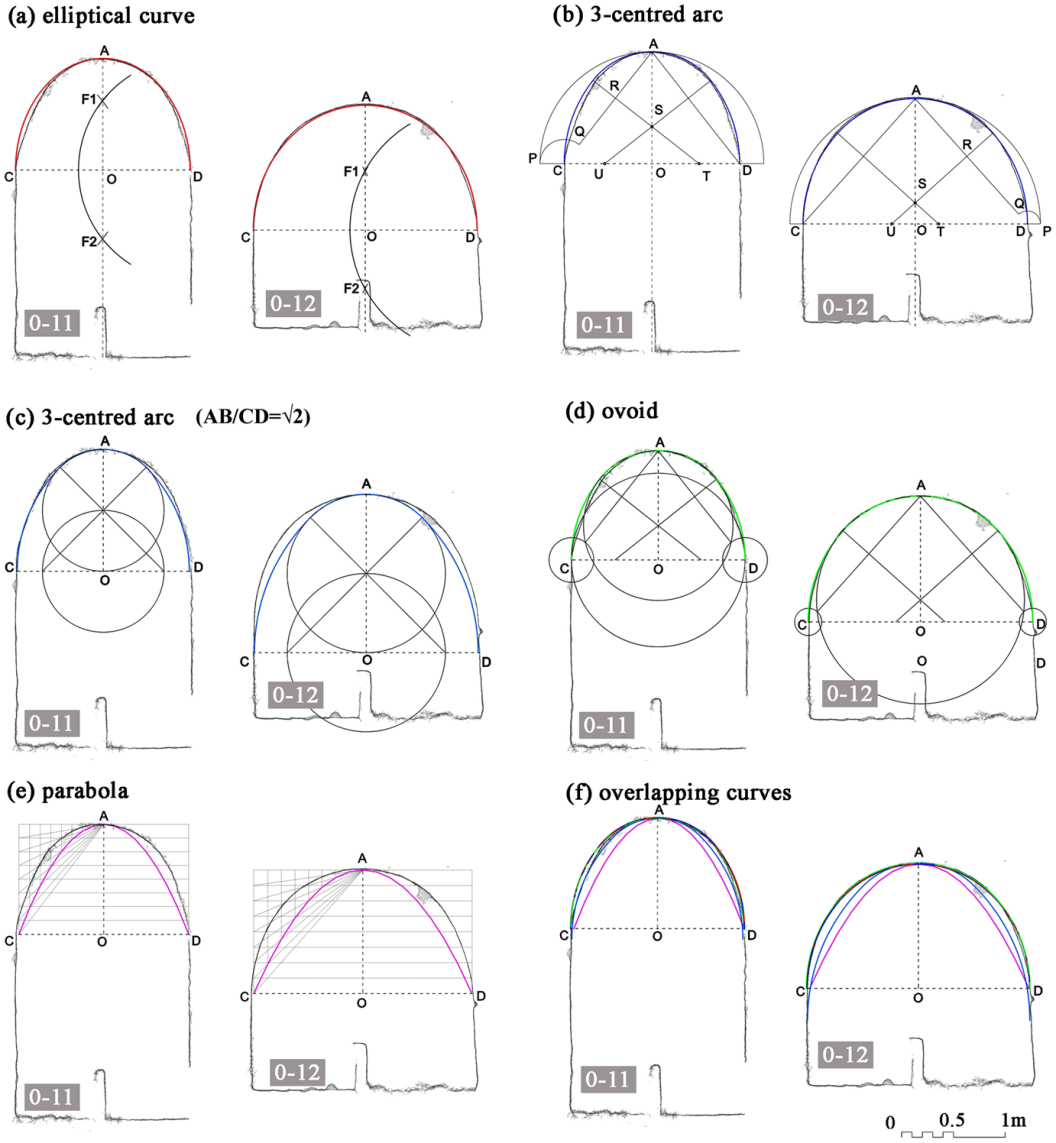
Profile of the vaults 0–13 and 0–14 (from section DD) to which the following geometric constructions have been superimposed: **a** ellipse (red profile); **b** generic three-centred arc (dark blue profile); **c** 3-centred arc in which AB/CD=√2 (light blue profile); **d** ovoid (green profile); **e** parabola (purple profile) (drawing by F. Fiorillo)

These tests were carried out on very thin vertical sections of the 3D point model of the Fort. Whenever it was possible to check, we noted that the profile of the vaults remained more or less constant along their length, and therefore that one vertical section for each vault would be sufficient to represent it. We superimposed all the geometric constructions listed in Fig. 4 to the profiles of all the visible vaults, and evaluated them one by one on the basis of the two chosen criteria. In order to summarise and present the results, we chose one pair of meaningful vaults, 0–13 and 0–14 from the vertical section D-D, which represent the two typical silhouettes: one narrower and elongated and the other wider and flatter (Fig. 9).

Despite the absence of a simple geometric method to draw a parabola, this shape was superimposed on the vertical sections of the vaults under study in order to visualize the constant and substantial discrepancy in comparison to the actual profiles. The graphic construction was based on the vertex A (corresponding to the point of the maximum height of the arc), the vertical axis of symmetry and the point C, corresponding to the vault impost on the wall (Fig. 9e).

Three-centred arcs (half of four-centred curves) show a marked similarity to the actual vaults. Of the three three-centred curves which have been taken into account, the ‘upper part’ of the ovoid (Fig. 9d) fits well (first criterion) but was discarded due to its extremely complicated geometric construction (second criterion). The shape of the narrower rooms fits particularly well the specific case of oval curve in which the proportion between the axes is equal to √2 (Fig. 9c) (Canciani 2015). An interesting aspect is that the circle defining the uppermost portion of the vault would have a radius of 1 cubit. This type of construction, however, once applied to the larger rooms, does not correspond to their shape as it falls too short on either side. We checked the ratio existing between the major and the minor axes in order to detect the presence of specific proportions, leading in turn to the adoption of specific geometric constructions (Dotto 2002), but no pattern could be detected. The generic three-centred arc fits well both types of vaults (Fig. 9b), but fails the second criterion: its geometric construction relies on points that fall outside the supporting walls of the vaults. In fact, this curve should be traced by connecting a first circumference with the center in S and radius SA, a second centred in T and radius TC and a third centred in U and radius UC (Fig. 9b) (Docci and Migliari 1992: 462–463). Therefore, tracing this profile on the short wall would be, in most of the cases, very uncomfortable, if not impossible.

**Fig. 10:**
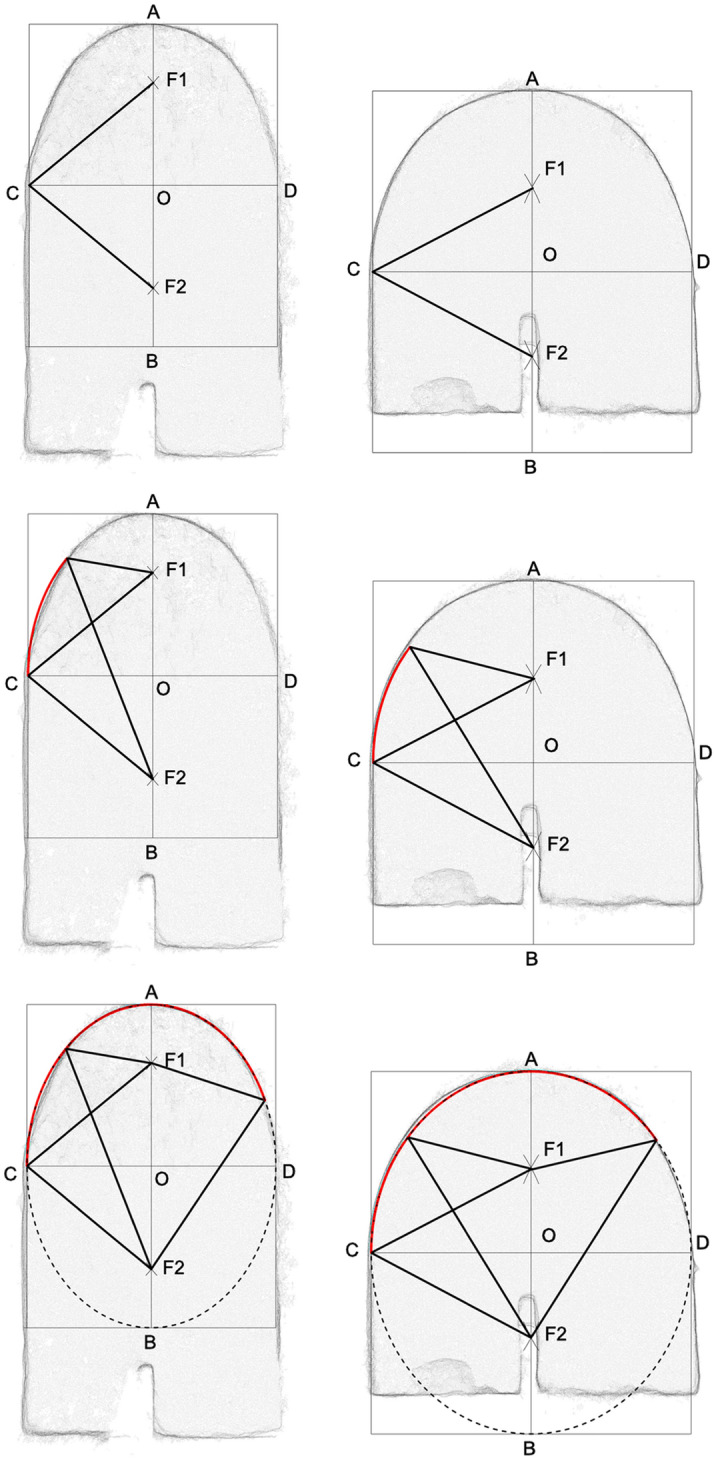
Profile of the vaults 0–13 and 0–14 (from section DD) showing the geometric construction of the elliptical curves drawn on the back wall before the construction of the corresponding vaults (drawing by F. Fiorillo)

The only curve that fits both the first and the second criteria is the ellipse (Fig. 9a). In this case, the major axis corresponds to twice the measure from the vault's impost to its maximum quota (AO). The minor axis CD is instead equal to the distance between the two walls of the room. Once these measures are known, it is possible to define the position of the two foci on the vertical axis by taking the vertical semi-axis as radius of an arc centred in C or D. The elliptical profile could be thus drawn on the back wall: the extremes of a rope (the length of which was twice the major axis) would be tied to two nails fixed in correspondence to the two foci (F1 and F2), stretched to the impost and then dragged upwards and then downwards on the other side to define the elliptical curve (Fig. 10) (Docci and Migliari 1992: 461).

## Constructional, Geometric and Metrological Considerations

The building method could thus be described as follows: for each level, the back wall (that is, the external wall of the building) was built vertically up to a height of at least 5 cubits, corresponding to the distance between floors. At every floor level, the thickness of the external wall diminished by progressively reducing the number of mudbricks constituting the wall (see Rossi and Fiorillo 2018: 384), thus creating a clear and visible step on the internal surface of the wall under construction; this could be used as reference line by the workmen.

**Fig. 11:**
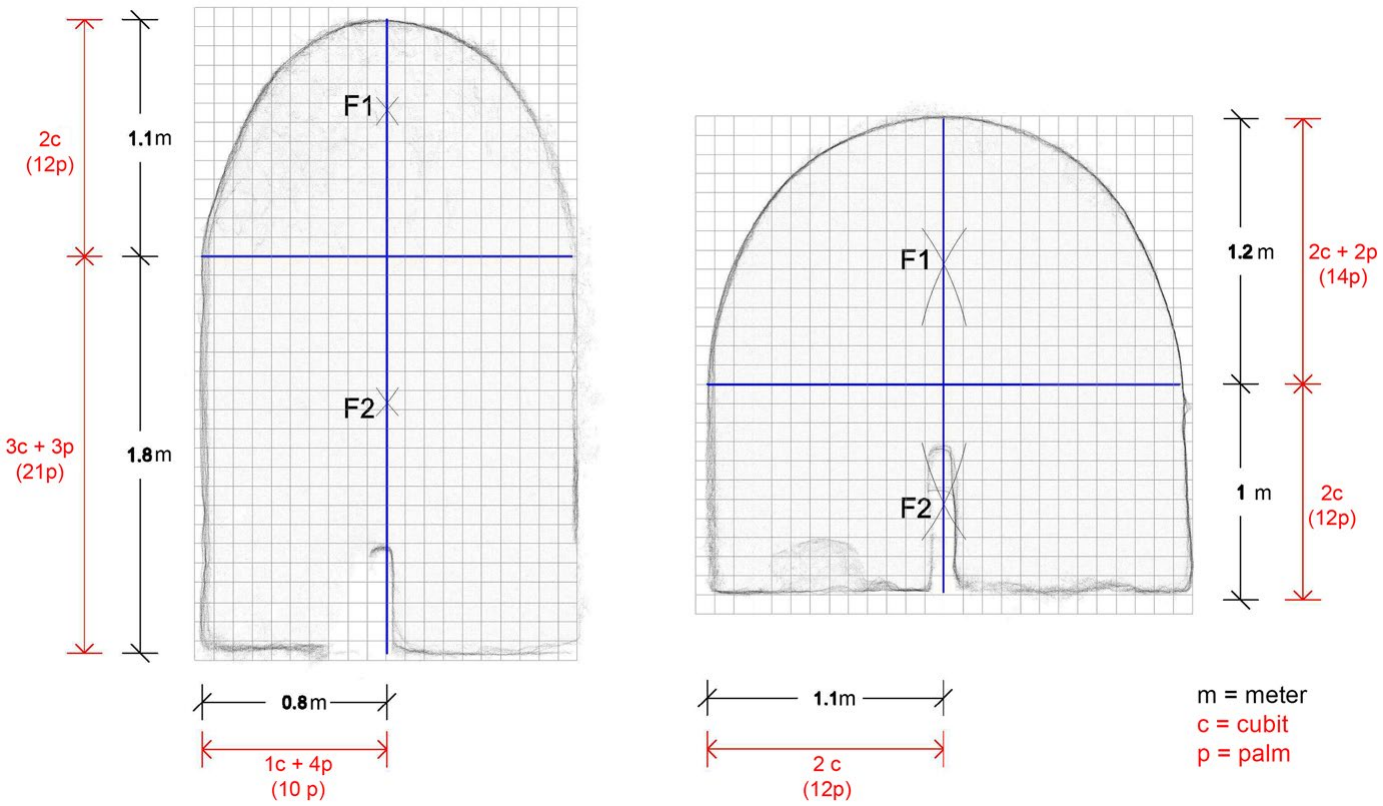
1 palm square grid superimposed on the vertical section of rooms 0–13 and 0–14 highlighting the difference in palms between the rise and half of the clear span of the two arches (drawing by F. Fiorillo)

The 1 cubit thick lateral walls of each room were built up to the height from which the vault was expected to spring. The height of these walls—that is the level of the impost of the vault—was probably established room by room on the basis of a precise consideration: as the vault’s profile was expected to be elongated, the workmen had to stop before the rise of the arch became equal to (or shorter than) half of the clear span. Superimposing a 1 palm grid on the vertical section of the rooms made it possible to establish that the difference between the two semi-axes measured either 1, 2 or 3 palms (the discrepancy being smaller in the larger rooms, Fig. 11). In the narrow rooms, the rise of the arches corresponds exactly or very closely to 2 cubits (the circle with a radius of 1 cubit would therefore simply highlight this dimension, and not be part of a geometric construction).

The elliptical curve was then drawn on the back wall using two nails and a rope as described above, and this curve would be used as reference point to start the construction of the growing portions of oblique arches.

Some vaults show a slight discrepancy in their mid-portion: the actual line of the vault runs lower than both the ellipse and all the three-centred arcs, but still far too high for the parabola (see Fig. 9f). This small difference might be explained in two ways: either the construction technique led the workmen to inadvertently modify the profile of the vault as it grew, or the weight of the filling resting on either side of the extrados determined a small deformation of the profile of the vault over the time.

## Conclusions

In general, the elongated shape of pitched-brick barrel vaults could be explained in one of two ways: as the casual result of an empirical constructional technique, or as the carefully planned result of a geometric construction; a third, intermediate way, is the possibility that a *simple* geometric construction was used to guide the hands of the workmen. The current study embraces this third option.

The results of this investigation can be applied to fresh analyses to be carried out at different scales. First of all, it could be applied on the rest of the building: once the ellipse has been identified as the inspiration for the design of the visible vaults, the discussion can be extended to the partial profiles that emerge out of the debris; this, in turn, can help to reconstruct the internal layout of the rooms that are currently inaccessible or that collapsed long ago. Then it could be applied to the rest of the Fortified Settlement: it is very likely that the construction technique was the same, as all the settlement was built at the same time. Considering the strong architectural similarities that characterise the network of forts that punctuate the Kharga Oasis, finding the same pattern in the other forts and in their accompanying settlements would not be surprising.

It will be then interesting to extend this discussion to the Late Roman settlements located in the Dakhla Oasis, the other half of the Oasis Magna, and then, with the due caution, to other contemporary settlements in other parts of Egypt, provided that precise and accurate surveys are available. Extending the analysis presented here to a wider corpus of vaulted buildings will hopefully offer fresh evidence to understand how the Roman and Egyptian technical and architectural knowledge combined in the Late Period not only in the Kharga Oasis, but in the entire province.
